# A Shh-Foxf-Fgf18-Shh Molecular Circuit Regulating Palate Development

**DOI:** 10.1371/journal.pgen.1005769

**Published:** 2016-01-08

**Authors:** Jingyue Xu, Han Liu, Yu Lan, Bruce J. Aronow, Vladimir V. Kalinichenko, Rulang Jiang

**Affiliations:** 1 Division of Developmental Biology, Cincinnati Children’s Hospital Medical Center, Cincinnati, Ohio, United States of America; 2 Division of Plastic Surgery, Cincinnati Children’s Hospital Medical Center, Cincinnati, Ohio, United States of America; 3 Division of Biomedical Informatics, Cincinnati Children’s Hospital Medical Center, Cincinnati, Ohio, United States of America; 4 Division of Pulmonary Biology, Perinatal Institute, Cincinnati Children’s Hospital Medical Center, Cincinnati, Ohio, United States of America; University of Southern California, UNITED STATES

## Abstract

Cleft palate is among the most common birth defects in humans. Previous studies have shown that Shh signaling plays critical roles in palate development and regulates expression of several members of the forkhead-box (Fox) family transcription factors, including Foxf1 and Foxf2, in the facial primordia. Although cleft palate has been reported in mice deficient in *Foxf2*, whether Foxf2 plays an intrinsic role in and how Foxf2 regulates palate development remain to be elucidated. Using Cre/loxP-mediated tissue-specific gene inactivation in mice, we show that Foxf2 is required in the neural crest-derived palatal mesenchyme for normal palatogenesis. We found that *Foxf2* mutant embryos exhibit altered patterns of expression of *Shh*, *Ptch1*, and *Shox2* in the developing palatal shelves. Through RNA-seq analysis, we identified over 150 genes whose expression was significantly up- or down-regulated in the palatal mesenchyme in *Foxf2*^*-/-*^ mutant embryos in comparison with control littermates. Whole mount *in situ* hybridization analysis revealed that the *Foxf2* mutant embryos exhibit strikingly corresponding patterns of ectopic *Fgf18* expression in the palatal mesenchyme and concomitant loss of *Shh* expression in the palatal epithelium in specific subdomains of the palatal shelves that correlate with where *Foxf2*, but not *Foxf1*, is expressed during normal palatogenesis. Furthermore, tissue specific inactivation of both *Foxf1* and *Foxf2* in the early neural crest cells resulted in ectopic activation of *Fgf18* expression throughout the palatal mesenchyme and dramatic loss of *Shh* expression throughout the palatal epithelium. Addition of exogenous Fgf18 protein to cultured palatal explants inhibited *Shh* expression in the palatal epithelium. Together, these data reveal a novel Shh-Foxf-Fgf18-Shh circuit in the palate development molecular network, in which Foxf1 and Foxf2 regulate palatal shelf growth downstream of Shh signaling, at least in part, by repressing *Fgf18* expression in the palatal mesenchyme to ensure maintenance of *Shh* expression in the palatal epithelium.

## Author Summary

Cleft lip and/or cleft palate (CL/P) are among the most common birth defects in humans, occurring at a frequency of about 1 in 500–2500 live births. The etiology and pathogenesis of CL/P are complex and poorly understood. Generation and analysis of mice carrying targeted null and conditional mutations in many genes have revealed that functional disruption of each of more than 100 genes could cause cleft palate. However, how these genes work together to regulate palate development is not well understood. In this study, we identify a novel molecular circuit consisting of two critical molecular pathways, the fibroblast growth factor (FGF) and Sonic hedgehog (SHH) signaling pathways, and the Forkhead family transcription factors Foxf1 and Foxf2, mediating reciprocal epithelial-mesenchymal signaling interactions that control palatogenesis. As mutations affecting each of multiple components of both the FGF and SHH signaling pathways have been associated with CL/P in humans, our results provide significant new insight into the mechanisms regulating palatogenesis and cleft palate pathogenesis.

## Introduction

The mammalian secondary palate develops from the oral side of the embryonic maxillary processes as a pair of outgrowths, which initially grow vertically to form the palatal shelves flanking the developing tongue. As development proceeds, the palatal shelves reorient to the horizontal position above the dorsum of the tongue, grow towards and subsequently fuse with each other at the midline to form the roof of the oral cavity. Genetic or environmental perturbations of any of these developmental processes, including palatal shelf growth, elevation and fusion, cause cleft palate, one of the most common congenital birth defects in humans [1–4].

Previous studies have shown that palatal shelf growth is regulated by reciprocal signaling interactions between the epithelium and the underlying neural crest-derived mesenchyme. At the onset of the palatal outgrowth, the secreted signaling molecule Sonic hedgehog (Shh) is expressed in the oral epithelium [5]. Shh is a mitogen and promotes cell proliferation in many embryonic and adult tissues [6]. Explant culture assays indicate that exogenous Shh protein stimulates palatal mesenchyme proliferation [7, 8]. Tissue-specific inactivation of the *Smoothened* (*Smo*) gene, which encodes a transmembrane protein required for transduction of the Shh signaling, in the early cranial neural crest cells resulted in complete absence of secondary palate structures in the *Smo*^*c/n*^*;Wnt1-Cre* mutant mice [9]. Moreover, tissue-specific inactivation of *Shh* in the oral epithelium or *Smo* in the early palatal mesenchyme resulted in defects in palatal shelf growth in the mutant mouse embryos [3, 8, 10]. Whereas the mechanism by which Shh signaling regulates palatal mesenchyme cell proliferation is incompletely understood, Shh signaling regulates palatal epithelial cell proliferation indirectly through, at least in part, activation of the fibroblast growth factor Fgf10 in the palatal mesenchyme [8]. Remarkably, in addition to regulating palatal epithelial cell proliferation, both Fgf10 and its epithelial receptor Fgfr2b are required for maintenance of *Shh* expression in the developing palatal epithelium [8]. Thus, Shh and Fgf10 signaling pathways function in a positive feedback loop to control palatal shelf growth [10].

In addition to its interaction with Fgf10 signaling, Shh signaling has also been shown to cooperate with Bmp signaling to regulate palatal shelf growth. In palatal explant culture assays, exogenous Shh protein induces *Bmp2* mRNA expression [7]. Tissue-specific inactivation of *Smo* in the palatal mesenchyme caused down-regulation of *Bmp2* expression in the anterior palatal mesenchyme [10], indicating that Shh signaling is required for maintenance of *Bmp2* expression during normal palatogenesis. Bmp signaling plays a critical role in anterior palatal shelf growth, as targeted deletion of *Bmpr1a*, encoding a type I receptor for Bmp ligands, in either the early neural crest or in the early palatal mesenchyme resulted in cleft of the anterior palate [11, 12]. Moreover, mice lacking the homeobox transcription factor Msx1 exhibit complete cleft palate that could be rescued by transgenic expression of *Bmp4* driven by the *Msx1* gene promoter [7]. During palatal shelf growth, the *Msx1*^*-/-*^ mutant mouse embryos showed reduced expression of *Bmp4* in the anterior palatal mesenchyme as well as reduced expression of *Shh* in the anterior palatal epithelium, in comparison with wildtype embryos [7]. Transgenic *Bmp4* expression in the anterior palatal mesenchyme was sufficient to restore *Shh* expression in the anterior palatal epithelium in the *Msx1*^*-/-*^ embryos, suggesting that Bmp4 acts downstream of Msx1 in the anterior palatal mesenchyme to maintain *Shh* expression in the anterior palatal epithelium [7].

The forkhead-box (Fox) family proteins form a large family of DNA-binding transcription factors [13, 14]. Through comparative transcriptional profiling of E10.5 embryonic head tissues of *Shh* mutant and control mouse embryos, Jeong et al. (2004) found that expression of several Fox family genes, including *Foxc2*, *Foxd1*, *Foxd2*, *Foxf1*, and *Foxf2*, in the neural crest derived facial mesenchyme was dependent on Shh signaling and suggested that these Fox family transcription factors might be key mediators of Hh pathway function in craniofacial development [9]. Both *Foxf1* and *Foxf2* are expressed in the developing palatal mesenchyme in wildtype mouse embryos and tissue-specific deletion of *Smo* also caused significant reduction in expression of *Foxf1* and *Foxf2* in the palatal mesenchyme [10, 15]. Foxf1 and Foxf2 display highly conserved amino acid sequences in the Forkhead DNA binding domain (100% identical in mouse and 97% identical in human FOXF subfamily) [16–18]. Whereas mouse embryos lacking Foxf1 function die during midgestation due to severe defects in the extraembryonic mesoderm [19], mice lacking Foxf2 die shortly after birth with a cleft palate phenotype [15]. Mutations in *FOXF2* have also been associated with cleft palate in humans [20]. However, how Foxf2 regulates palate development remains to be elucidated.

In this study, we show that *Foxf1* and *Foxf2* exhibit partially overlapping patterns of expression during palate development, with *Foxf2* expressed more broadly than *Foxf1* along the anterior-posterior axis of the developing palatal shelves. By using *Cre/loxP* mediated conditional gene inactivation, we demonstrate that the Foxf1 and Foxf2 transcription factors act partly redundantly to control palatal shelf growth through a novel Foxf-Fgf18-Shh regulatory circuit.

## Results

### *Foxf2* is required in the neural crest-derived palatal mesenchyme for normal palatal shelf growth

**Fig 1:**
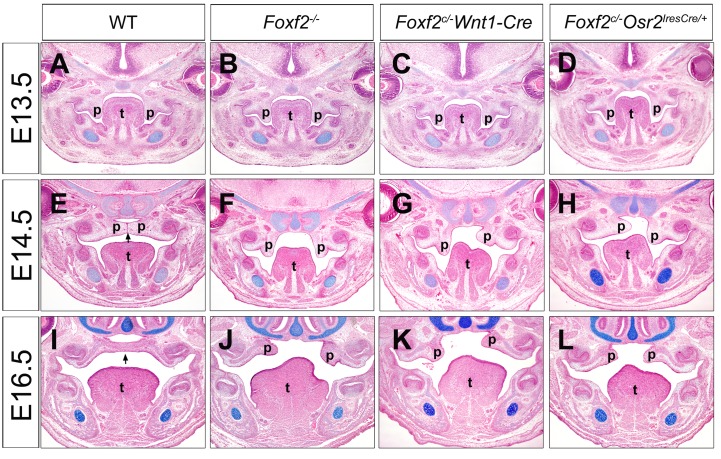
Histological analysis of palate developmental defects in *Foxf2* mutant mouse embryos. Representative frontal sections of wildtype (A, E, I), *Foxf2*^*-/-*^ (B, F, J), *Foxf2*^*c/-*^*Wnt1-Cre* (C, G, K), and *Foxf2*^*c/-*^*Osr2*^*IresCre/+*^ (D, H, L) embryos at E13.5 (A-D), E14.5 (E-H) and E16.5 (I-L). p, palatal shelf; t, tongue.

Wang et al. (2003) reported that *Foxf2*-deficient mice die shortly after birth and exhibit cleft palate. Although Wang et al. (2003) detected *Foxf2* mRNA expression in the developing palatal shelves in wildtype mouse embryos, they found that *Foxf2* mRNAs were more abundantly expressed in the muscle layers of the developing tongue and suggested that the cleft palate defect in *Foxf2*^*-/-*^ mice might be secondary to defects in tongue movement because the tongue did not properly descend in the mutant embryos [15]. To determine whether Foxf2 plays an intrinsic role in palate development, we generated mice with tissue-specific inactivation of *Foxf2* in the cranial neural crest lineage or in the developing palatal mesenchyme using mice carrying a loxP-flanked *Foxf2* conditional allele (*Foxf2*^*c*^) [21]. The *Wnt1-Cre* transgenic mice have been shown to exhibit Cre recombinase activity in the premigratory neural crest cells that give rise to most of the non-muscle mesenchyme in the craniofacial tissues [22–24]. On the other hand, the *Osr2*^*IresCre/+*^ mice exhibit highly specific expression of the Cre recombinase in the *Osr2*-expressing palatal mesenchyme cells and with no Cre activity in the muscle cells of the developing tongue [10, 25]. We found that both *Foxf2*^*c/-*^*Wnt1-Cre* and *Foxf2*^*c/-*^*Osr2*^*IresCre/+*^ mice have complete penetrance of cleft palate and many of the mutant embryos showed failure of palatal shelf elevation, similar to the cleft palate phenotype in mice with constitutive inactivation of the *Foxf2* gene (*Foxf2*^*-/-*^), (Fig 1). These results suggest that Foxf2 function is required in the palatal mesenchyme for normal palatogenesis.

**Fig 2:**
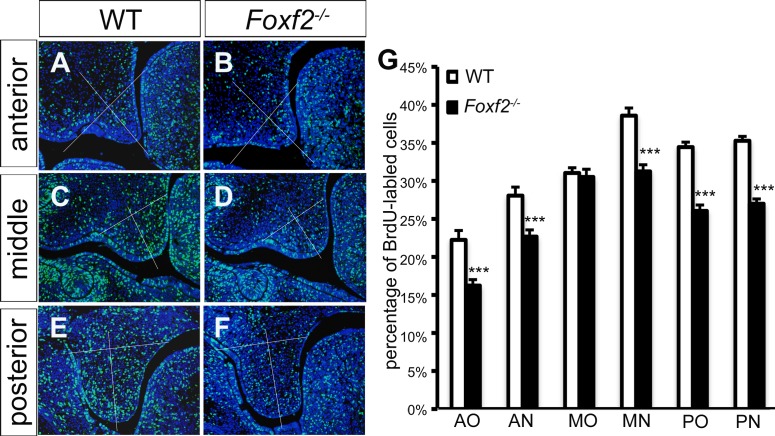
Analysis of cell proliferation in the developing palate in E13.5 *Foxf2*^*-/-*^ and wildtype embryos. (A-F) Representative images of sections through the anterior (A, B), middle (C, D) and posterior (E, F) regions of the palate in wildtype (A, C, E) and *Foxf2*^*-/-*^ mutant (B, D, F) embryos showing distribution of immunostained BrdU-labeled nuclei (green). Sections were counterstained with DAPI (blue). White line divides the palatal shelf into oral and nasal sides for cell counts. (G) The percentage of BrdU-labeled cells in the E13.5 palatal mesenchyme (***p<0.001). AO, oral half of the anterior region; AN, nasal half of the anterior region; MO, Oral half of the middle region; MN, nasal half of the middle region; PO, oral half of the posterior region; PN, nasal half of the posterior region.

To investigate whether Foxf2 function is required for palatal shelf growth, we performed BrdU incorporation assays in E13.5 embryos. Since the developing palatal shelves exhibit morphological and molecular heterogeneity along the anterior-posterior and oral-nasal axes [1], we analyzed the BrdU-labeling index separately for the oral and nasal halves of the palatal shelves in each of the anterior, middle, and posterior sub-regions, with the middle region corresponding to that flanked by the maxillary first molar tooth buds (Fig 2A–2F). We found that *Foxf2*^*-/-*^ mutant embryos exhibited significant reduction in cell proliferation in the anterior region, nasal half of the middle region, and the posterior region of the palatal mesenchyme (Fig 2G). These data confirm an intrinsic role for Foxf2 in palate development.

### Differential molecular effects of Foxf2 deficiency along the anterior-posterior axis of the developing palatal shelves

**Fig 3:**
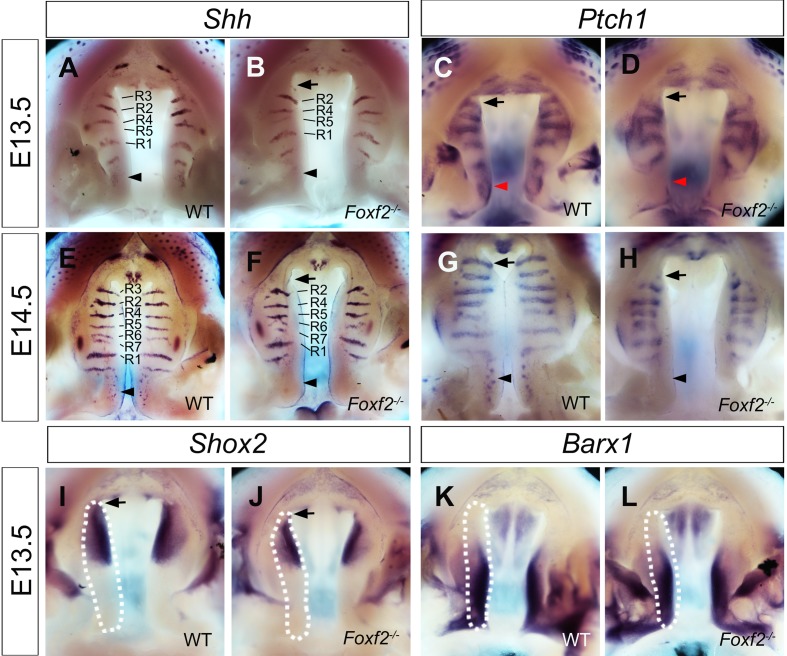
Comparison of patterns of *Shh*, *Ptch1*, *Shox2*, and *Barx1* mRNA expression in *Foxf2*^*-/-*^ mutant and wildtype control embryos. (A, B, E, F) Expression patterns of *Shh* mRNAs in the developing palatal shelves in wildtype (A, E) and *Foxf2*^*-/-*^ mutant (B, F) embryos at E13.5 (A, B) and E14.5 (E, F). (C, D, G, H) Expression patterns of *Ptch1* mRNAs in the developing palatal shelves in wildtype (C, G) and *Foxf2*^*-/-*^ mutant (D, H) embryos at E13.5 (C, D) and E14.5 (G, H). In specific anterior (arrows) and posterior (arrow heads) subdomains of the palatal shelves, *Shh* and *Ptch1* expression is dramatically downregulated in *Foxf2*^*-/-*^ mutant embryos. R1-R7 mark the individual ruga in the order of their formation. (I-L) Expression patterns of *Shox2* (I, J) and *Barx1* (K, L) mRNAs in the palatal shelves in wildtype (I, K) and *Foxf2*^*-/-*^ mutant (J, L) embryos at E13.5. White dashes demarcate the palatal shelf on the right side. Arrows in I and J point to the anterior region of the palatal shelves, where the expression of *Shox2* mRNAs is reduced in *Foxf2*^*-/-*^ mutant embryos.

To gain insight into the molecular mechanisms mediating Foxf2 function in palate development, we analyzed whether the expression patterns of a number of genes known to play critical roles in palate development were altered in the *Foxf2*^*-/-*^ mutant palatal shelves. Shh signaling has been shown to regulate palatal shelf growth and *Shh* mRNA expression marks the palatal rugae, the epithelial ridges that form in specific spatiotemporal patterns on the oral surface of the palatal shelves during palatal outgrowth [10, 26, 27]. Thus, the whole mount *Shh* mRNA expression pattern has been used as a valuable molecular marker for analysis of palatal shelf growth or patterning defects in mutant mouse studies [12, 27–29]. In comparison with the *Shh* mRNA expression pattern in the palatal epithelium in wildtype embryos (Fig 3A and 3E), the *Foxf2*^*-/-*^ mutant embryos exhibited specific loss of the most anterior *Shh* mRNA expression domain that corresponds to Ruga-3 (Fig 3B and 3F) at E13.5 and E14.5. *Shh* mRNA expression in the posterior palatal epithelium was also dramatically downregulated in the *Foxf2*^*-/-*^ mutant embryos in comparison with the wildtype littermates (Fig 3A, 3B, 3E and 3F). Corresponding to the region-specific loss of *Shh* mRNA expression, expression of *Ptch1*, a well-known direct transcriptional target of canonical Hedgehog signaling [30–33], was specifically downregulated in the Ruga-3 region as well as in the posterior palate in the *Foxf2*^*-/-*^ mutant embryos in comparison with the wildtype littermates (Fig 3C, 3D, 3G and 3H).

We examined the expression patterns of *Shox2* and *Barx1*, which mark the anterior and posterior halves of the developing palatal mesenchyme, respectively [27, 34, 35]. The level of *Shox2* mRNA expression was reduced, especially in the anterior-most region of the palatal shelves, in *Foxf2*^*-/-*^ mutant embryos in comparison with the wildtype littermates (Fig 3I and 3J). In contrast, the pattern and levels of expression of *Barx1* in the developing palatal shelves were not obviously altered in *Foxf2*^*-/-*^ mutant embryos in comparison with the wildtype littermates (Fig 3K and 3L).

Previous studies showed that the Msx1-Bmp4 and Fgf10 signaling pathways regulate palatal shelf growth as well as *Shh* mRNA expression in the palatal epithelium [7, 8, 28]. We examined whether palatal expression of these genes was affected by *Foxf2* deficiency. By *in situ* hybridization analyses, however, we didn’t detect obvious differences in expression of *Msx*1, *Bmp4*, and *Fgf10*, respectively, in the developing palatal shelves between *Foxf2*^*-/-*^ mutant and wildtype littermates (S1A–S1J Fig). Moreover, we performed quantitative RT-PCR analysis of manually microdissected palatal shelves from E13.5 wildtype, *Foxf2*^*+/-*^, and *Foxf2*^*-/-*^ littermates but did not detect any significant differences in expression levels of *Bmp4*, *Fgf10*, and *Msx1* mRNAs, respectively, between the samples of different genotypes.

**Fig 4:**
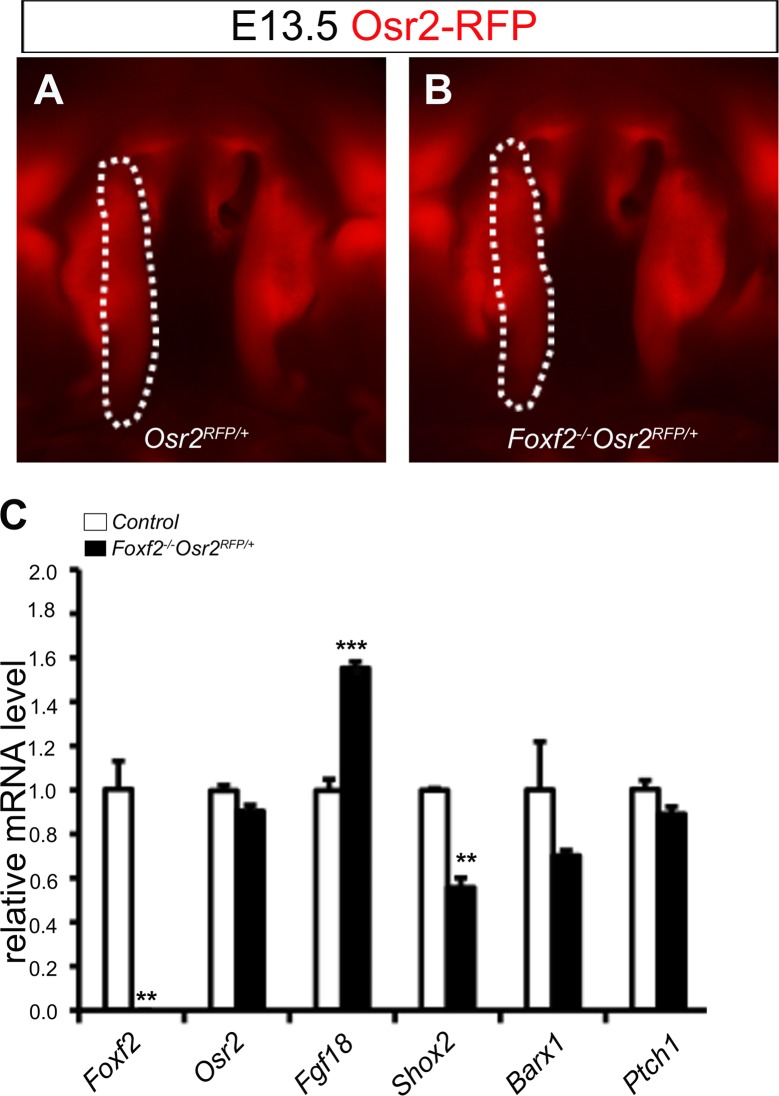
Differential gene expression analysis of palatal mesenchyme cell in control and *Foxf2* mutant embryos by using RNAseq and real time RT-PCR assays. (A, B) The patterns of RFP expression in the *Osr2*^*RFP/+*^ (A) and *Foxf2*^*-/-*^*Osr2*^*RFP/+*^ (B) palatal shelves are comparable. White dashes demarcate the palatal shelf on the right side. (C) Real time RT-PCR analysis of the levels of expression of *Foxf2*, *Osr2*, *Fgf18*, *Shox2*, *Barx1*, and *Ptch1* mRNAs in E13.5 palatal mesenchyme cells in control and *Foxf2*^*-/-*^*Osr2*^*RFP/+*^ embryos (**p<0.01, ***p<0.001).

To gain a better understanding of the molecular mechanisms mediating Foxf2 function in palate development, we compared the transcriptome expression profiles of E13.5 *Foxf2*^*-/-*^ and control palatal mesenchyme by using RNA-seq analysis. To facilitate RNA-seq analysis of palatal mesenchyme cells, and since it has been shown that *Foxf2* and *Osr2* are expressed in a similar oral-to-nasal gradient pattern in the developing palatal mesenchyme at E13.5 [10], we took advantage of the *Osr2*^*RFP/+*^ knockin mice for isolation of palatal mesenchyme cells using fluorescence-activated cell sorting (FACS). We crossed *Foxf2*^*+/-*^ mice with *Osr2*^*RFP/+*^ mice to generate the *Foxf2*^*+/-*^*Osr2*^*RFP/+*^ mice and then set up timed mating of the *Foxf2*^*+/-*^ female mice with *Foxf2*^*+/-*^*Osr2*^*RFP/+*^ male mice. We verified that RFP expression in the developing palate was not affected by *Foxf2*-deficiency (Fig 4A and 4B). We harvested embryos at E13.5, microdissected the palatal shelves from each RFP-positive embryo, and isolated the RFP-positive palatal mesenchyme cells by FACS. Following identification of the embryo genotypes, we performed RNA-seq analysis of the FACS-isolated palatal mesenchyme from *Foxf2*^*-/-*^*Osr2*^*RFP/+*^, *Foxf2*^*+/-*^*Osr2*^*RFP/+*^ and *Foxf2*^*+/+*^*Osr2*^*RFP/+*^embryos, respectively. Differential expression analysis of the RNA-seq data identified 155 genes whose expression was up- or down-regulated by more than 1.5-fold in the *Foxf2*^*-/-*^*Osr2*^*RFP/+*^ palatal mesenchyme in comparison with both *Foxf2*^*+/-*^*Osr2*^*RFP/+*^ and *Foxf2*^*+/+*^*Osr2*^*RFP/+*^ samples (S1 Table). Among these, *Fgf18*, which encodes a member of the fibroblast growth factor family ligands, was up-regulated by more than 2-fold in *Foxf2*^*-/-*^*Osr2*^*RFP/+*^ mutant palatal mesenchyme compared with the control littermates (S1 Table). Subsequent quantitative real-time RT-PCR analysis validated the significantly increased expression of *Fgf18* in the *Foxf2*^*-/-*^ mutant palatal mesenchyme (Fig 4C). Consistent with results from whole mount *in situ* hybridization, expression of *Shox2* was significantly decreased whereas expression of *Osr2* and *Barx1* were not significantly changed in the *Foxf2*^*-/-*^ palatal mesenchyme in comparison with the control samples (Fig 4C).

**Fig 5:**
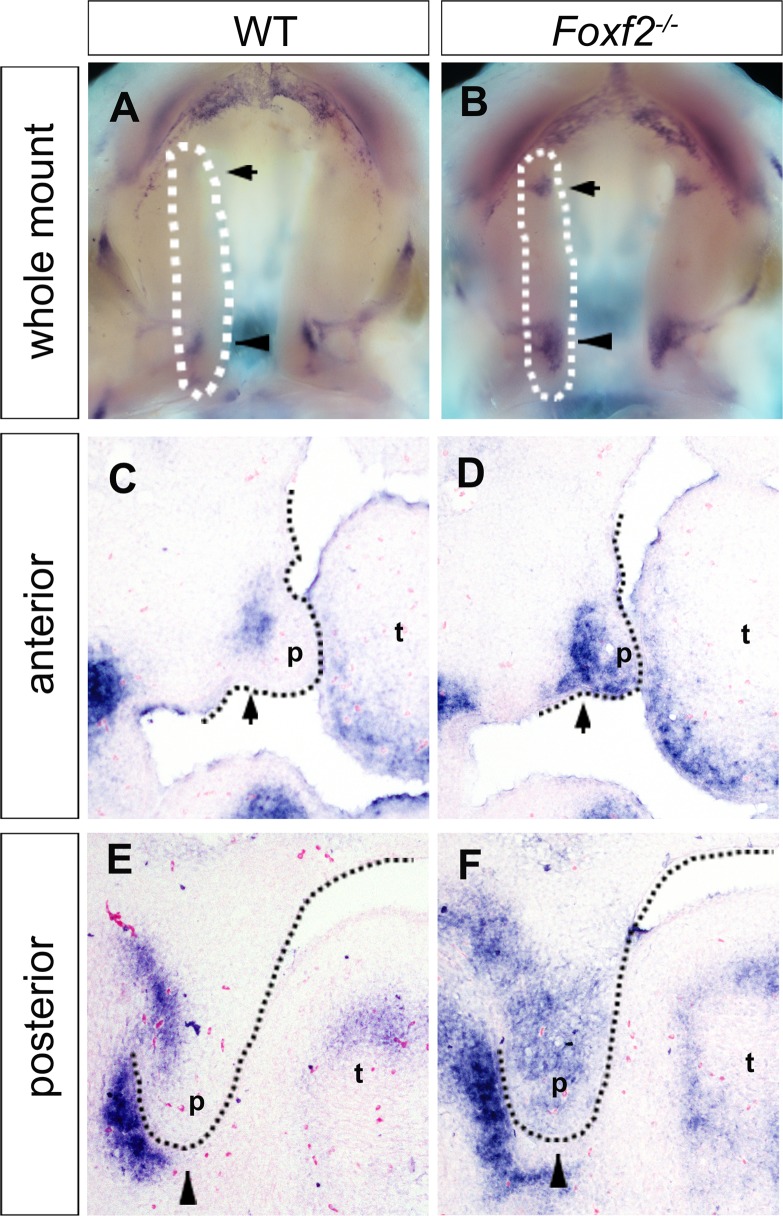
Comparison of expression of *Fgf18* mRNAs in the palatal shelves in wildtype and *Foxf2*^*-/-*^ mutant embryos. (A, B) Whole-mount *in situ* hybridization detection of *Fgf18* mRNAs in the developing palatal shelves in wildtype (A) and *Foxf2*^*-/-*^ mutant (B) embryos at E13.5. White dashes demarcate the palatal shelf on the right side. Note that *Fgf18* is ectopically expressed in specific anterior (arrow) and posterior (arrow head) subdomains of *Foxf2*^*-/-*^ mutant palatal shelves. (C-F) Frontal sections showing expression of *Fgf18* mRNAs in the anterior (C, D) and posterior (E, F) regions of the developing palatal shelves in wildtype (C, E) and *Foxf2*^*-/-*^ mutant (D, F) embryos at E13.5. p, palatal shelf; t, tongue.

We further compared the patterns of *Fgf18* expression in the *Foxf2*^*-/-*^ and littermate control embryos by whole mount *in situ* hybridization. Strikingly, we found that *Fgf18* mRNAs were ectopically expressed in specific anterior and posterior sub-regions of the developing palatal shelves in the *Foxf2*^*-/-*^ mutant embryos (Fig 5A and 5B) that correspond to where expression of both *Shh* and *Ptch1* was dramatically downregulated in the *Foxf2*^*-/-*^ mutant embryos in comparison with the control embryos (compare Fig 5A and 5B with Fig 3A–3H). Further *in situ* hybridization analysis of serial coronal sections through the E13.5 palatal shelves confirmed ectopic expression of *Fgf18* mRNAs in the palatal mesenchyme in the specific anterior and posterior regions in the *Foxf2*^*-/-*^ mutant embryos while *Fgf18* mRNA expression in the wildtype littermates was restricted to the mesenchyme cells at the hinge region of the palatal shelves (Fig 5C–5F).

### The molecular effects of Foxf2 deficiency on palate development correspond to distinct expression patterns of *Foxf1* and *Foxf2* in the developing palatal shelves

**Fig 6:**
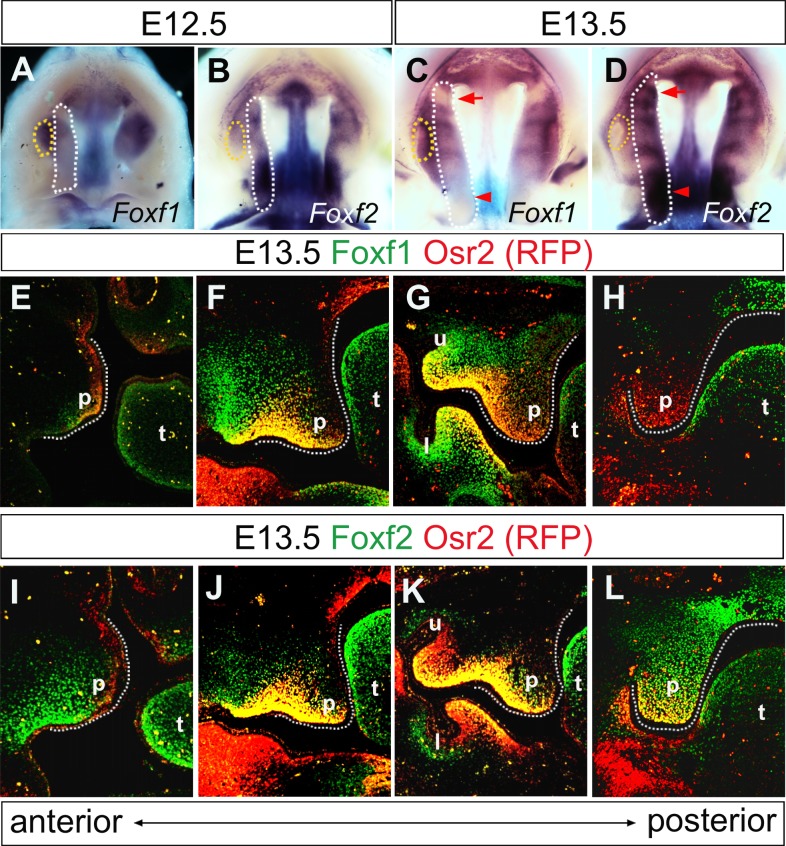
The patterns of *Foxf1* and *Foxf2* expression during palate development. (A-D) Whole-mount *in situ* hybridization detection of *Foxf1* (A, C) and *Foxf2* (B, D) mRNAs in the developing palate shelves in E12.5 (A, B) and E13.5 (C, D) mouse embryos. White dashes demarcate the palatal shelf on the right side. Yellow dashes mark the molar tooth germ. (E-H) Immunofluorescent staining of Foxf1 (green) and RFP (red) on sections from the anterior to posterior regions of the palatal shelves in E13.5 *Osr2*^*RFP/+*^ embryos. (I-L) Immunofluorescent staining of Foxf2 (green) and RFP (red) on sections in E13.5 *Osr2*^*RFP/+*^ embryos. p, palatal shelf; t, tongue; l, lower molar; u, upper molar.

To understand why the *Fox2*^*-/-*^ mutant embryos exhibit region-specific changes in gene expression profiles along the anterior-posterior axis of the developing palatal shelves, we analyzed and compared the patterns of expression of *Foxf2* and *Foxf1* during palate development. From E12.5 to E13.5, *Foxf1* mRNA expression was restricted to the middle region of developing palatal shelves, with the strongest level of expression detected in the molar tooth germs (Fig 6A and 6C). In contrast, *Foxf2* mRNA expression was detected throughout the anterior-posterior axis of the developing palatal shelves, with the posterior region of the palatal shelves exhibiting higher levels of expression than the anterior region at both E12.5 and E13.5 (Fig 6B and 6D).

We further analyzed the distribution of the Foxf1 and Foxf2 proteins in the developing palatal shelves by immunofluorescent detection in *Osr2*^*RFP/+*^ knockin mouse embryos, which express the RFP reporter from the endogenous *Osr2* locus that exhibits an oral-to-nasal gradient pattern of expression in the developing palatal mesenchyme [36]. At E13.5, Foxf1 protein is expressed at moderate levels in the mid-anterior region of the palatal mesenchyme while it is expressed very weakly in the anterior-most region and absent in the posterior region of the developing palatal shelves (Fig 6E–6H). Foxf2 protein is distributed throughout the anterior-posterior axis of the palatal mesenchyme, but with different patterns along the oral-nasal axis in the anterior versus posterior regions (Fig 6I–6L). In the anterior region up to the level of the molar tooth germs, Foxf2 protein distribution exhibited an oral-to-nasal gradient, with highest levels in the mesenchyme immediately underneath the palatal epithelium at the oral side (Fig 6I, 6J and 6K). In the palatal region posterior to the molar tooth germs, Foxf2 protein is expressed at high levels throughout the oral-nasal axis of the palatal mesenchyme (Fig 6L). Neither Foxf1 nor Foxf2 protein was detected in the palatal epithelium. These data indicate that expression of *Foxf1* and of *Foxf2* are differentially regulated during palate development. Remarkably, the regions where expression of *Fgf18*, *Shh*, and *Ptch1* is significantly altered in the palatal shelves in *Foxf2*^*-/-*^ embryos, compared with the wildtype littermates, correspond to the palatal regions where *Foxf2*, but not *Foxf1*, is expressed during palate development in wildtype embryos, suggesting that Foxf1 might complement Foxf2 function in the middle region of the developing palatal shelves in *Foxf2*^*-/-*^ mutant embryos.

### *Foxf1* and *Foxf2* act partly redundantly to represses expression of *Fgf18* in the developing palatal mesenchyme

**Fig 7:**
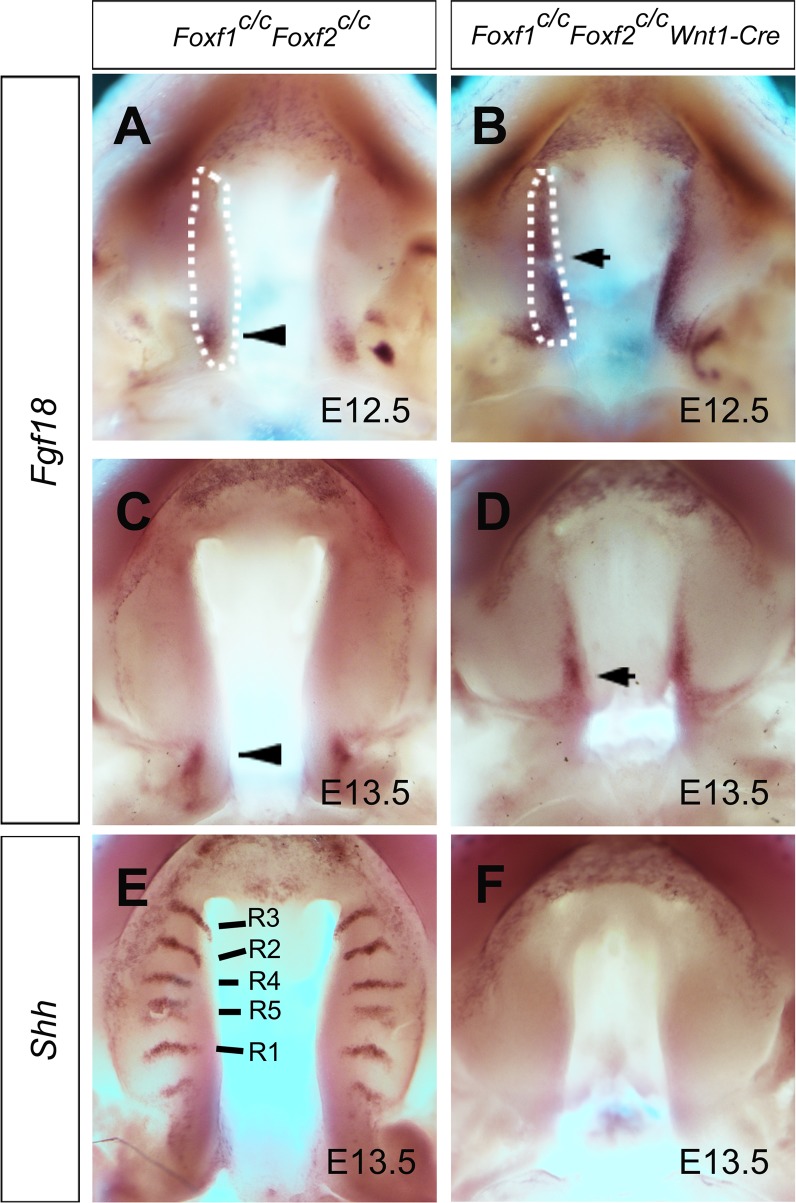
Comparison of expression of *Fgf18* and *Shh* mRNAs in the palatal shelves in *Foxf1*^*c/c*^*Foxf2*^*c/c*^ control and *Foxf1*^*c/c*^*Foxf2*^*c/c*^*Wnt1-Cre* mutant embryos. (A, B) Whole-mount *in situ* hybridization detection of *Fgf18* mRNAs in the developing palatal shelves in *Foxf1*^*c/c*^*Foxf2*^*c/c*^ control (A, C) and *Foxf1*^*c/c*^*Foxf2*^*c/c*^*Wnt1-Cre* mutant (B, D) embryos at E12.5 (A, B) and E13.5 (C, D). White dashes demarcate the palatal shelf on the right side. Arrowhead (A, C) and arrow (B, D) point to the *Fgf18* expression domains in the control and mutant palatal shelves, respectively. (E, F) Whole-mount *in situ* hybridization detection of *Shh* mRNAs in the developing palatal shelves in *Foxf1*^*c/c*^*Foxf2*^*c/c*^ control (E) and *Foxf1*^*c/c*^*Foxf2*^*c/c*^*Wnt1-Cre* mutant (F) embryos at E13.5. Note that expression of *Shh* is lost in *Foxf1*^*c/c*^*Foxf2*^*c/c*^*Wnt1-Cre* mutant palatal shelves.

Since *Foxf1*^*-/-*^ mutant mouse embryos die during midgestation prior to palate morphogenesis [19], which prevents a direct analysis of the role of Foxf1 in palate development in these mice, we generated and analyzed *Foxf1*^*c/c*^*Wnt1-Cre* mouse embryos in which *Foxf1* is tissue-specifically inactivated in the neural crest derived craniofacial mesenchyme. Whereas *Foxf1*^*c/+*^*Wnt1-Cre* mice appear normal, *Foxf1*^*c/c*^*Wnt1-Cre* mice are born with cleft palate (S2A and S2B Fig). Histological analysis of E16.5 embryos showed that the *Foxf1*^*c/c*^*Wnt1-Cre* mutant embryos exhibit failure of palatal shelf elevation (S2C and S2D Fig). However, in contrast to the *Foxf2*^*-/-*^ mutant embryos, the patterns of expression of *Fgf18* and *Shh* mRNAs in the developing palatal shelves were not significantly altered in the *Foxf1*^*c/c*^*Wnt1-Cre* mutant embryos in comparison with the control littermates (S3A–S3D Fig). To directly test the hypothesis that the region-specific molecular effects of *Foxf2* deficiency on palate development is due to functional complementation by *Foxf1*, we generated *Foxf1*^*c/c*^*Foxf2*^*c/c*^*Wnt1-Cre* embryos and examined the patterns of *Fgf18* and *Shh* expression by whole mount *in situ* hybridization. Remarkably, although the *Foxf1*^*c/c*^*Foxf2*^*c/c*^*Wnt1-Cre* embryos have smaller palatal shelves compared with the control littermates, they exhibit ectopic *Fgf18* expression specifically throughout the palatal shelves at E12.5 and E13.5, compared with the highly restricted pattern of *Fgf18* mRNA expression in control embryos (Fig 7A–7D). Analysis by *in situ* hybridization of serial coronal sections confirmed ectopic expression of *Fgf18* mRNAs from anterior to posterior regions of the palatal mesenchyme in the *Foxf1*^*c/c*^*Foxf2*^*c/c*^*Wnt1-Cre* mutant embryos at E12.5 (S4 Fig). Furthermore, we found that *Shh* mRNA expression in the palatal epithelium was dramatically lost throughout the anterior-posterior axis of the palatal shelves in *Foxf1*^*c/c*^*Foxf2*^*c/c*^*Wnt1-Cre* mutant embryos in comparison with the control littermates (Fig 7E and 7F). In addition, although the palatal shelves in the *Foxf1*^*c/c*^*Foxf2*^*c/c*^*Wnt1-Cre* embryos still exhibited *Shox2* expression in the anterior region and *Barx1* in the posterior region, both the domain and level of *Shox2* expression were dramatically reduced in comparison with that in the control littermates (S5 Fig). These results suggest that *Foxf1* and *Foxf2* act partly redundantly to regulate *Fgf18* and *Shox2* mRNA expression in the palatal mesenchyme and act indirectly to maintain *Shh* expression in the palatal epithelium during early palate development.

### Exogenous Fgf18 protein inhibits *Shh* expression in the developing palate

**Fig 8:**
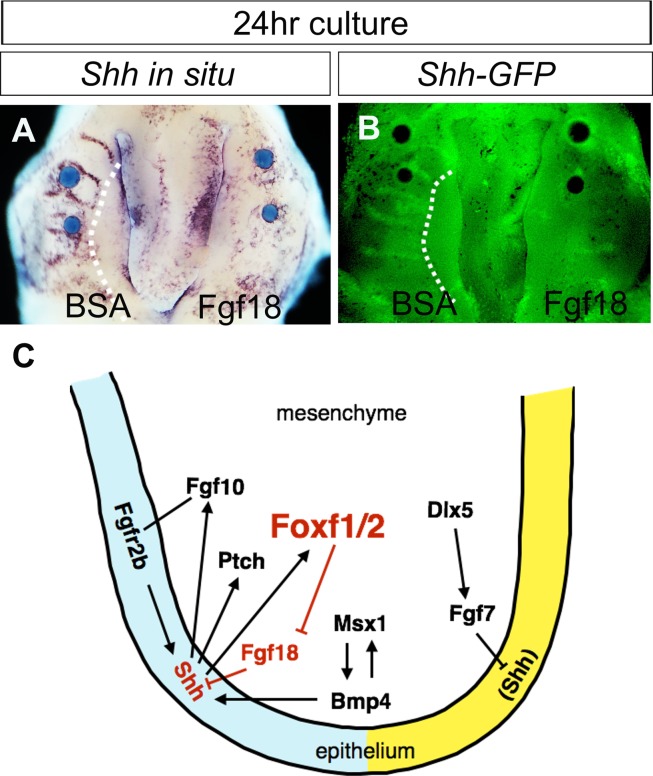
Regulation of *Shh* and *Fgf18* expression during palate development. (A) Whole-mount *in situ* detection of *Shh* mRNAs in wildtype embryonic palatal explants treated with BSA beads (left side) and Fgf18 beads (right side). (B) The patterns of GFP expression in *Shh*^*GFP/+*^ embryonic palatal explants treated with BSA beads (left side) and Fgf18 beads (right side). Note that *Shh* expression is inhibited by Fgf18 beads. (C) A model depicting the molecular regulation of *Foxf1*, *Foxf2*, *Fgf18*, and *Shh* expression in the developing palatal shelf. Regulation of *Shh* expression by Fgf10/Fgfr2b, Msx1/Bmp4, and Dlx5/Fgf7 pathways is summarized from References 8, 7, and 28, respectively.

We next investigated whether the ectopic *Fgf18* expression in the palatal shelves could account for loss of *Shh* expression in the palatal epithelium in the *Foxf2*^*-/-*^ and *Foxf1*^*c/c*^*Foxf2*^*c/c*^*Wnt1-Cre* mutant embryos. We placed Fgf18-soaked beads on the oral side of E13.0 palatal shelves in explant culture and examined effects of Fgf18 protein on *Shh* expression in the palatal epithelium after 24 hours of culture. As shown in Fig 8, application of Fgf18 protein caused a dramatic down-regulation of *Shh* gene expression in the palatal epithelium whereas the BSA-soaked beads did not have any obvious effect, as detected by *in situ* hybridization of *Shh* mRNA expression (Fig 8A) and by detection of GFP reporter expressed from the *Shh*^*GFP*^ allele in *Shh*^*GFP/+*^ embryonic palates (Fig 8B). These results, together with the corresponding domains of ectopic *Fgf18* expression in the palatal mesenchyme and of loss of *Shh* expression in the palatal epithelium in the *Foxf2*^*-/-*^ and *Foxf1*^*c/c*^*Foxf2*^*c/c*^*Wnt1-Cre* mutant embryos, respectively, indicate that Foxf1 and Foxf2 regulate palatogenesis, at least in part, through repressing *Fgf18* expression to maintain Shh signaling to stimulate palatal shelf growth.

## Discussion

Shh signaling plays critical roles in the growth and patterning of the developing palatal shelves [8–10, 37, 38]. Disruption of the function of Shh, Smo, or the transcriptional effectors Gli2 or Gli3, during palatal development each causes cleft palate in the mutant mice [8, 10, 39, 40], indicating that Shh signaling regulates palate development through Gli mediated transcriptional regulation. Whereas direct target genes of Gli transcription factors have not been specifically isolated from the developing palatal shelves, previous studies demonstrated that expression of the *Foxf1* and *Foxf2* in the craniofacial and palate mesenchyme dependent on Shh signaling [9, 10]. By using high-throughput sequencing of immunoprecipitated Gli3-bound chromatin fragments from developing mouse embryonic tissues, Hoffmann et al. recently showed that Gli3 bound to cis-regulatory sequences close to multiple *Fox* family genes, including *Foxf1* [41]. Analysis of one of the Gli3-binding *Foxf1* enhancer sequences driving lacZ reporter expression in transgenic mouse embryos showed specific enhancer activity in multiple Hedgehog responding tissues, indicating that *Foxf1* is a direct target of Gli-mediated transcriptional regulation [41]. Whereas *Foxf1*^*-/-*^ and *Foxf2*^*-/-*^ mutant mouse embryos exhibit distinct developmental defects, with early embryonic lethality of *Foxf1*^*-/-*^ mutant embryos and cleft palate in *Foxf2*^*-/-*^ embryos, which match the distinct embryonic expression patterns of these two genes [15, 19, 42], *Foxf1* and *Foxf2* also exhibit overlapping expression in multiple developing tissues and *Foxf1*^*+/-*^*Foxf2*^*+/-*^ compound heterozygous mouse embryos exhibit defects in gut and cardiac septation [41, 43], indicating that Foxf1 and Foxf2 act synergistically in many developmental processes. Although *Foxf2*^*-/-*^ mutant mice have been reported to exhibit cleft palate phenotype, how Foxf2 and Foxf1 regulate palate development have not been documented. In this study, we found that Foxf1 and Foxf2 function partly redundantly to regulate palate development. Through transcriptional profiling, we found that Foxf1 and Foxf2 act to repress the expression of *Fgf18* in the developing palatal mesenchyme. We found that both *Foxf2*^*-/-*^ and *Foxf1*^*c/c*^*Foxf2*^*c/c*^*Wnt1-Cre* mutant embryos exhibit loss of *Shh* expression in the palatal epithelial regions specifically corresponding to the domains of ectopic *Fgf18* expression and that exogenous Fgf18 protein inhibited *Shh* expression in the palatal epithelium in palatal explant culture. These data identify a novel Foxf-Fgf18-Shh negative feedback loop regulating Shh signaling in palate development (Fig 8C).

### Differential expression and functional overlap between Foxf1 and Foxf2 in palate development

Although expression of both *Foxf1* and *Foxf2* mRNAs in the developing craniofacial and palatal mesenchyme depends on Shh signaling [9, 10], we detected *Foxf2* mRNA expression throughout the anterior-posterior axis of the developing palatal mesenchyme but *Foxf1* mRNA expression is absent from specific anterior and posterior sub-regions of the developing palatal shelves, suggesting that other molecular pathways converge with the Shh signaling pathway to differentially regulate *Foxf1* and *Foxf2* expression during palate development. Consistent with this hypothesis, Hoffmann et al. (2014) recently identified a *Foxf1* cis-regulatory element that bound both Gli1 and the T-box transcription factor Tbx5 in the developing heart tissues and that Gli1 and Tbx5 synergistically activated transcription from this cis-regulatory element [41]. In embryonic lung explant culture assays, *Foxf1* mRNA expression in the lung mesenchyme was shown to be induced by Shh and repressed by Bmp4 [44]. We recently reported that Bmp4 is expressed in an anterior and a posterior subdomain of the developing palatal mesenchyme [29]. Future studies will determine whether *Foxf1* expression during palate development is directly and antagonistically regulated by Shh and Bmp4 signaling.

### Foxf2 plays an intrinsic role in palate development

Wang et al (2003) first showed cleft palate defect in the *Foxf2*^*-/-*^ mutant mice, but suggested that the cleft palate phenotype might be secondary to defects in tongue muscle development because *Foxf2* mRNAs are abundantly expressed in the muscle layers of the developing tongue in wildtype mouse embryos, and because they did not detect a significant difference in cell proliferation in the developing palate at E13.5 and E15.5 using BrdU labeling. Our analysis of BrdU labeling of the palatal mesenchyme detected significant reduction in palatal mesenchyme proliferation at E13.5, at the peak of palatal shelf growth. The discrepancy in these findings are most likely due to differences in the BrdU labeling procedure and in data analysis. We injected the pregnant mice intraperitonially with 50 μg/g body weight BrdU and harvested the embryos one hour later. This procedure resulted in labeling of up to 40% of the palatal mesenchyme cells in E13.5 wildtype embryos. In contrast, Wang et al. (2003) injected the pregnant mice intraperitonially with 100 μg/g body weight and sacrificed the injected animals 2 hours later. At E13.5, this procedure might have saturated labeling of the palatal mesenchyme cells. In addition, since the developing palatal shelves have both morphological and molecular heterogeneity [1], we recorded the percentage of BrdU-labeled palatal mesenchyme cells on serial sections throughout the palatal shelves and analyzed the data separately in the six regions along the anterior-posterior and oral-nasal axes. It is not clear how Wang et al. analyzed the BrdU-labeling data. Given the heterogeneity of the palatal mesenchyme and our finding that the molecular effects of *Foxf2*-deficeincy on the developing palatal shelves are most pronounced in the anterior and posterior domains where *Foxf2*, but not *Foxf1*, is expressed during normal palatogenesis, it is possible that simple analysis of the BrdU-labeling index in the middle region of palatal shelves would not find significant differences in the mutant and control embryos. Indeed, our data show that cell proliferation index is not significantly different in the mid-oral portion of the developing palatal shelves in the *Foxf2*^*-/-*^ and control littermates (Fig 2G). The differential effects of loss of Foxf2 function on palatal cell proliferation along the anterior-posterior axis is mostly likely due to partial functional compensation by Foxf1 in the middle region of the developing palatal shelves. The fact that the *Foxf1*^*c/c*^*Foxf2*^*c/c*^*Wnt1-Cre* compound mutant embryos exhibit only rudimentary palatal shelves supports the conclusion that Foxf1 and Foxf2 act partly redundantly to control palatal shelf growth.

Our conclusion that Foxf2 plays an intrinsic role in palate development is also supported by the molecular effects of *Foxf2*-deficiency on palatal gene expression. The domain-specific changes in *Fgf18* and *Shh* expression in the developing palatal shelves in the *Foxf2*^*-/-*^ mutant embryos correlated with the lack of *Foxf1* expression in the anterior and posterior regions of the palatal mesenchyme. Moreover, we found that *Foxf1*^*c/c*^*Foxf2*^*c/c*^*Wnt1-Cre* embryos exhibit ectopic *Fgf18* mRNA expression specifically throughout the palatal mesenchyme and loss of *Shh* mRNA expression throughout the palatal epithelium. Thus, similar to their functions in gut and heart development in which Foxf1 and Foxf2 exhibit synergistic effects [41, 43], Foxf1 and Foxf2 could partly complement for each other’s function during palate development in the regions where they exhibit overlapping expression.

Whereas the cell proliferation and molecular studies clearly demonstrate an intrinsic role for Foxf2 in palate development, both *Foxf2*^*c/-*^*Wnt1-Cre* and *Foxf2*^*c/-*^*Osr2*^*IresCre/+*^ embryos exhibit defects in palatal shelf elevation and abnormal tongue shape (Fig 1G and 1H). Whereas the *Foxf2*^*c/-*^*Wnt1-Cre* embryos are expected to have loss of *Foxf2* function throughout the neural crest-derived craniofacial mesenchyme, including the non-muscle connective tissues in the tongue, which could cause a primary defect in tongue development, the *Osr2*^*IresCre/+*^ embryos exhibit only limited Cre activity in a subset of tongue mesenchyme cells directly underlying the tongue epithelium [10, 25]. However, it is possible that loss of Foxf2 function in the small population of tongue mesenchyme cells directly underlying the epithelium also perturbs epithelial-mesenchymal interactions during tongue development. Thus, whether disruption of Foxf2 function in the developing tongue could secondarily affect palatal shelf elevation remains to be investigated by generation and analysis of mice with tissue-specific inactivation of *Foxf2* in the developing tongue.

### A Shh-Foxf-Fgf18-Shh regulatory circuit in the molecular network controlling palate development

Our RNA-seq analysis of *Foxf2*^*-/-*^ and control embryonic palatal mesenchyme revealed that Foxf2-deficiency significant affected the expression of over 150 genes in the developing palate. Our *in situ* hybridization analysis revealed the striking pattern of ectopic *Fgf18* expression in the *Foxf2*^*-/-*^ mutant palate, which correlated well with the unique domains of *Foxf2*, but not *Foxf1*, expression in the developing palatal shelves in wildtype embryos. This, together with the data that *Fgf18* is expressed throughout the palatal mesenchyme in the *Foxf1*^*c/c*^*Foxf2*^*c/c*^*Wnt1-Cre* embryos suggests that *Fgf18* is a direct target gene repressed by the Foxf transcription factors. Although *Shh* expression in the palatal epithelium also exhibits domain-specific loss in the *Foxf2*^*-/-*^ mutant embryos, the loss of *Shh* expression in the palatal epithelium in the *Foxf1*^*c/c*^*Foxf2*^*c/c*^*Wnt1-Cre* mutant embryos, in which *Foxf1* and *Foxf2* are specifically inactivated in the mesenchyme, indicate that the Foxf transcription factors indirectly regulate *Shh* expression. Together with the findings that exogenous Fgf18 protein inhibited *Shh* expression in palatal explant culture and our previously reported data that expression of *Foxf1* and *Foxf2* in the palatal mesenchyme depends on Shh signaling [10], these results identify a novel negative feedback loop controlling *Shh* expression during palate development.

Fgf18 belongs to the fibroblast growth factor family of ligands, which consists of 22 members and signal through alternatively spliced forms of tyrosine kinase receptors encoded by four distinct genes, *Fgfr1*, *Fgfr2*, *Fgfr3*, and *Fgfr4* [45, 46]. Previous studies have implicated Fgf7 and Fgf10 in the regulation of *Shh* expression during palate development [8, 28]. Whereas Fgf7 and Fgf10 share high amino acid sequence homology and both signal exclusively through the Fgfr2b receptor in epithelial tissues, they elicit distinct and sometime opposite cellular responses in developmental tissues as well as in cell culture assays [8, 28, 47, 48]. During palate development, *Fgf7* and *Fgf10* exhibit complementary expression patterns in the developing palatal mesenchyme, with *Fgf7* mRNAs preferentially expressed in the nasal side and *Fgf10* mRNAs restricted to the oral side of the palatal mesenchyme [8, 28]. Mice lacking Fgf10 or Fgfr2b exhibit cleft palate, with loss of Shh expression in the developing palatal epithelium [8]. In palatal explant culture assays, exogenous Fgf10 protein induced, whereas exogenous Fgf7 protein repressed, *Shh* mRNA expression, suggesting that Fgf7 antagonizes Fgf10 function to restrict Shh expression to the oral side of the palatal epithelium [8, 28]. Although the mechanism underlying the opposite effects of Fgf7 and Fgf10 on *Shh* expression during palate development is not known, Francavilla et al. (2013) recently reported that Fgf10 specifically induced rapid phosphorylation of the tyrosine (Y)-734 residue on Fgfr2b, which led to the receptor recycling and enhanced and prolonged Fgfr signaling, whereas Fgf7 led to rapid degradation of the receptors [48]. It is plausible that inhibition of *Shh* expression in the palatal explant by exogenous Fgf7 could be mediated by Fgf7-induced Fgfr2b degradation, as Fgfr2b function is required for maintenance of *Shh* expression in the palatal epithelium [8]. It is possible that the ectopically expressed Fgf18 in the *Foxf2*^*-/-*^ and *Foxf1*^*c/c*^*Foxf2*^*c/c*^*Wnt1-Cre* mutant palatal mesenchyme might also cause reduction in *Shh* expression in the palatal epithelium by inducing rapid Fgfr2b degradation. However, *in vitro* studies and prediction from crystal structures suggested that Fgf18 lacks affinity for Fgfr2b [49, 50]. On the other hand, Fgf18 has been shown to bind Fgfr3c and the cysteine-rich Fgf receptor [51, 52]. The detailed molecular mechanism involving Fgf18-mediated regulation of *Shh* expression during palate development requires further investigation. Interestingly, mice lacking Fgf18 function exhibit high penetrance of cleft palate [53, 54]. Moreover, genome-wide association studies of cleft lip and palate in humans have shown disease association with the *FGF18* locus [55]. Thus, further investigation of the role and molecular mechanisms involving Fgf18 in palate development will directly improve our understanding of the genetic basis and molecular mechanisms of cleft palate pathogenesis in humans.

## Materials and Methods

### Mouse strains

The *Foxf1*^*c/c*^, *Foxf2*^*c/c*^, *Wnt1-Cre* and *Osr2*^*IresCre/+*^ mice have been described previously [21, 22, 25, 56]. *Osr2*^*RFP/+*^ (JAX stock #010986) and *Shh*^*GFP/+*^ (JAX stock #005622) mice were obtained from the Jackson Laboratory. The *Osr2*^*IresCre*^ and *Osr2*^*RFP/+*^ mice were maintained by crossing to C57BL/6J mice. The *Wnt1Cre* mice were maintained by crossing with CD1 (Charles river) females. *Foxf1*^*c/c*^ and *Foxf2*^*c/c*^ mice were maintained by intercrossing homozygotes. Noon of the day a vaginal plug was identified was designated as embryonic day (E) 0.5. This study was performed in strict accordance with the recommendations in the Guide for the Care and Use of Laboratory Animals by the National Institutes of Health. The animal use protocol was approved by the Institutional Animal Care and Use Committee of Cincinnati Children’s Hospital Medical Center (Permit Number IACUC2013-0036).

### Histology and immunofluorescent staining

For histological analysis, embryos were dissected at desired stages from timed pregnant mice, fixed in 4% paraformaldehyde (PFA), dehydrated through an ethanol series, embedded in paraffin, sectioned at 7μm thickness, and stained with alcian blue followed by hematoxylin and eosin.

Immunofluorescent staining of paraffin sections was performed following standard protocols. Antibodies used are: rabbit anti-RFP (MBL international, PM005), Goat anti-Foxf1 (R&D, AF4798), and sheep anti-Foxf2 (R&D, AF6988).

### Cell proliferation assays

To determine cell proliferative activity in the developing palatal shelves, timed pregnant mice were injected intraperitoneally with BrdU (5 mg/ml stock solution, 10 μl/g body weight) (Sigma-Aldrich). Embryos were harvested 1 hour after injection, fixed with 4% PFA, paraffin embedded and sectioned at 7 μm. The BrdU-labeling index was defined as the number of BrdU-positive nuclei relative to total nuclei, which was co-stained by DAPI. The cell proliferation data were recorded from seven sections from each of the anterior, middle and posterior regions of each palatal shelf, and also analyzed separately for the oral and nasal halves of each region of the palatal shelves in each embryo. Data from two independent litters, each containing two wildtype and two *Foxf2*^*-/-*^ embryos, were used for statistical analysis.

### *In situ* hybridization

Whole mount and section *in situ* hybridization was performed as previously described [36, 57]. At least three embryos of each genotype were hybridized to each probe and only probes that detected consistent patterns of expression in all samples were considered as valid results.

### Palatal shelf explant culture and bead implantation

Palatal shelf explant culture and bead implantation experiments were carried out using a previously described protocol with minor modification [28]. Briefly, Timed pregnant mice were sacrificed on post-coital day 13.0 (E13.0). The embryonic maxillary processes with the secondary palatal shelves were manually microdissected and cultured in BGJb medium supplementary with 10 U/ml penicillin/ streptomycin (Invitrogen), 50 mM transferrin (Sigma) and 150 μg/ml ascorbic acid (Sigma). For bead implantation, Affi-Gel blue agarose beads (BioRad) were soaked in recombinant Fgf18 proteins (1mg/ml, Peprotech), or BSA (1mg/ml) as control. Tissues were harvested after 24 hours of culture at 37°C at an atmosphere of 5% CO2 and 100% humidity and fixed in 4% paraformaldehyde for whole mount *in situ* hybridization experiments.

### Fluorescence activated cell sorting and RNA-seq

The palatal shelves of E13.5 embryos from *Foxf2*^*+/-*^ females crossed with *Foxf2*^*+/-*^ *Osr2*^*RFP/+*^ males were manually microdissected and digested with trypsin-EDTA solution (Invitrogen) at 37°C for 4 minutes. After inactivation of trypsin with DMEM containing 10% FBS, cells were dissociated by pipetting. The dissociated palatal cells were resuspended in PBS with 2% FBS and 10 mM EDTA, and filtered through a 40 μm nylon cell strainer (BD Falcon, 352340). RFP^+^ cells were isolated using BD FACSAria II.

FACS-isolated RFP^+^ palatal mesenchyme cells from two E13.5 *Foxf2*^*-/-*^*Osr2*^*RFP/+*^ embryos and one *Foxf2*^*+/-*^*Osr2*^*RFP/+*^ and one *Osr2*^*RFP/+*^ littermates were used for RNA-seq experiment. *Foxf2*^*+/-*^*Osr2*^*RFP/+*^ and *Osr2*^*RFP/+*^ samples were used as controls. Sequencing libraries were generated by using Illumina Nextera DNA Sample Prep kit and sequenced using Illumina HisEq 2000. Sequence reads were mapped to the reference mouse genome (mm9) using Bowtie. Single-end reads were aligned using Tophat. RNA-seq data were then analyzed using Strand NGS software, with the reads per kilobase exon per million mapped sequences value calculated for each RefSeq gene for relative levels of gene expression. For analyses of differential expression, the fold-change cutoff was set at 1.5 or higher. P value less than 0.05 from the Audic Claverie test was considered statistically significant, with Benjamini–Hochberg false discovery rate multiple testing correction [58]. The original RNA-seq data files from this study have been deposited into the National Center for Biotechnology Information Gene Expression Omnibus (NCBI GEO) database (accession number GSE67015).

### Real-time RT-PCR

First-strand cDNAs were synthesized using SuperScript First-Strand Synthesis System (Invitrogen, 11904–018). Primers for specific transcripts were designed for real-time RT-PCR (SYBR). β-Actin was used as internal control in each reaction. Real-time PCR was performed using a Bio-Rad CFX96 Real-Time System using conditions recommended by the manufacturer. Each reaction was performed in duplicate. The quantity of each mRNA was first determined using a standard curve method and normalized to the internal control. The primers used for real-time RT-PCR are listed in S2 Table.

### Statistical analysis

All results were presented as mean ± SEM. All statistical analyses were done using Excel software. Two-tailed Student’s t tests were used for comparisons between two groups. P value less than 0.05 was considered significant.

## Supporting Information

S1 Table Differential expression analysis of the RNA-seq data in the *Foxf2*^*-/-*^*Osr2*^*RFP/+*^ palatal mesenchyme in comparison with the control palatal mesenchyme. (XLSX)

S2 Table Primers used in real-time RT-PCR assay. (DOCX)

S1 Fig Comparison of *Msx1*, *Bmp4*, and *Fgf10* mRNA expression patterns in wildtype and *Foxf2*^*-/-*^ mutant embryos. (A-F) Whole-mount *in situ* hybridization detection of *Msx1* (A, B), *Bmp4* (C, D), and *Fgf10* (E, F) mRNAs in the developing palatal shelves in wildtype (A, C, E) and *Foxf2*^*-/-*^ mutant (B, D, F) embryos at E13.5. White dash lines indicate the palate region. (G-J) Frontal sections showing expression of *Fgf10* mRNA in the anterior (G, H) and middle (I, J) regions of the developing palate in wildtype (G, I) and *Foxf2*^*-/-*^ mutant (H, J) embryos at E13.5. p, palatal shelf; t, tongue.
(TIF)

S2 Fig Analysis of palate development defects in *Foxf1*^*c/c*^*Wnt1-Cre* mutant mouse embryos. (A, B) Ventral view of stained skeletal preparations of *Foxf1*^*c/+*^*Wnt1-Cre* (A) and *Foxf1*^*c/c*^*Wnt1-Cre* (B) neonatal skulls. Arrowheads indicate palatal processes of the palatine bones that have fused to each other in the *Foxf1*^*c/+*^*Wnt1-Cre* mice (A) but are absent in the *Foxf1*^*c/c*^*Wnt1-Cre* mice, exposing the presphenoid bone (marked with an asterisk) underneath (B). (C, D) Representative frontal sections from developing palatal shelves of *Foxf1*^*c/+*^*Wnt1-Cre* (C), and *Foxf1*^*c/c*^*Wnt1-Cre* (D) embryos, at E16.5. p, palatal shelf; t, tongue.
(TIF)

S3 Fig Comparison of expression of *Fgf18* and *Shh* mRNAs in the palatal shelves in *Foxf1*^*c/+*^*Wnt1-Cre* and *Foxf1*^*c/c*^*Wnt1-Cre* mutant embryos. (A, B) Whole-mount *in situ* hybridization detection of *Fgf18* mRNAs in the developing palatal shelves in *Foxf1*^*c/c*^ (A) and *Foxf1*^*c/c*^*Wnt1-Cre* mutant (B) embryos at E13.5. (C, D) Whole-mount *in situ* hybridization detection of *Shh* mRNAs in the developing palatal shelves in *Foxf1*^*c/c*^ (C) and *Foxf1*^*c/c*^*Wnt1-Cre* mutant (D) embryos at E13.5.
(TIF)

S4 Fig Comparison of expression of *Fgf18* mRNAs in *Foxf1*^*c/c*^*Foxf2*^*c/c*^ and *Foxf1*^*c/c*^*Foxf2*^*c/c*^*Wnt1-Cre* mutant embryos. Frontal sections showing expression of *Fgf18* mRNA in the anterior (A, B), middle (C, D) and posterior (E, F) regions of the developing palate in *Foxf1*^*c/c*^*Foxf2*^*c/c*^ (A, C, E) and *Foxf1*^*c/c*^*Foxf2*^*c/c*^*Wnt1-Cre* mutant (B, D, F) embryos at E12.5. p, palatal shelf.
(TIF)

S5 Fig Comparison of *Shox2 and Barx1* mRNA expression patterns in *Foxf1*^*c/c*^*Foxf2*^*c/c*^ and *Foxf1*^*c/c*^*Foxf2*^*c/c*^*Wnt1-Cre* mutant embryos. (A-D) Whole-mount *in situ* hybridization detection of *Shox2* mRNAs in the developing palatal shelves in *Foxf1*^*c/c*^*Foxf2*^*c/c*^ (A, C) and *Foxf1*^*c/c*^*Foxf2*^*c/c*^*Wnt1-Cre* mutant (B, D) embryos at E12.5 (A, B) and E13.5 (C, D). (E-H) Whole-mount *in situ* hybridization detection of *Barx1* mRNAs in the developing palatal shelves in *Foxf1*^*c/c*^*Foxf2*^*c/c*^ (E, G) and *Foxf1*^*c/c*^*Foxf2*^*c/c*^*Wnt1-Cre* mutant (F, H) embryos at E12.5 (E, F) and E13.5 (G, H).
(TIF)
