# Supplementary material for: A Shh-Foxf-Fgf18-Shh Molecular Circuit Regulating Palate Development
Source: PLoS Genet. 2016 Jan 8;12(1):e1005769. doi: 10.1371/journal.pgen.1005769 (PMC4712829; doi:10.1371/journal.pgen.1005769)
Supplement: S2 Table — (DOCX) [file pgen.1005769.s002.docx]

**S2 table. Primers used in real-time RT-PCR assay.**

| **Primer** | **Sequence (5’-3’)** |
| --- | --- |
| Foxf2-F | agcatgtcttcctactcgttg |
| Foxf2-R | tctttcctgtcgcacact |
| Osr2-F | ttgctcattcacgagaggac |
| Osr2-R | tcccacactcctgacatttg |
| Fgf18-F | agtggagacagataccttcgg |
| Fgf18-R | gtacttggcagacatcaggg |
| Shox2-F | cccactatccagacgctttc |
| Shox2-R | acctttgtgaagttgattttcctg |
| Barx1-F | cctccgattttgataccacgt |
| Barx1-R | gtcgcaccgtattcactgag |
| Ptch1-F | tcaggcaatacgaagcacag |
| Ptch1-R | caagggaggctgatgtct |
| Actin-F | GAGAAGATCTGGCACCACACC |
| Actin-R | GCATACAGGGACAGCACAGC |
