# Supplementary figures and images for: A Shh-Foxf-Fgf18-Shh Molecular Circuit Regulating Palate Development
Source: PLoS Genet. 2016 Jan 8;12(1):e1005769. doi: 10.1371/journal.pgen.1005769 (PMC4712829; doi:10.1371/journal.pgen.1005769)

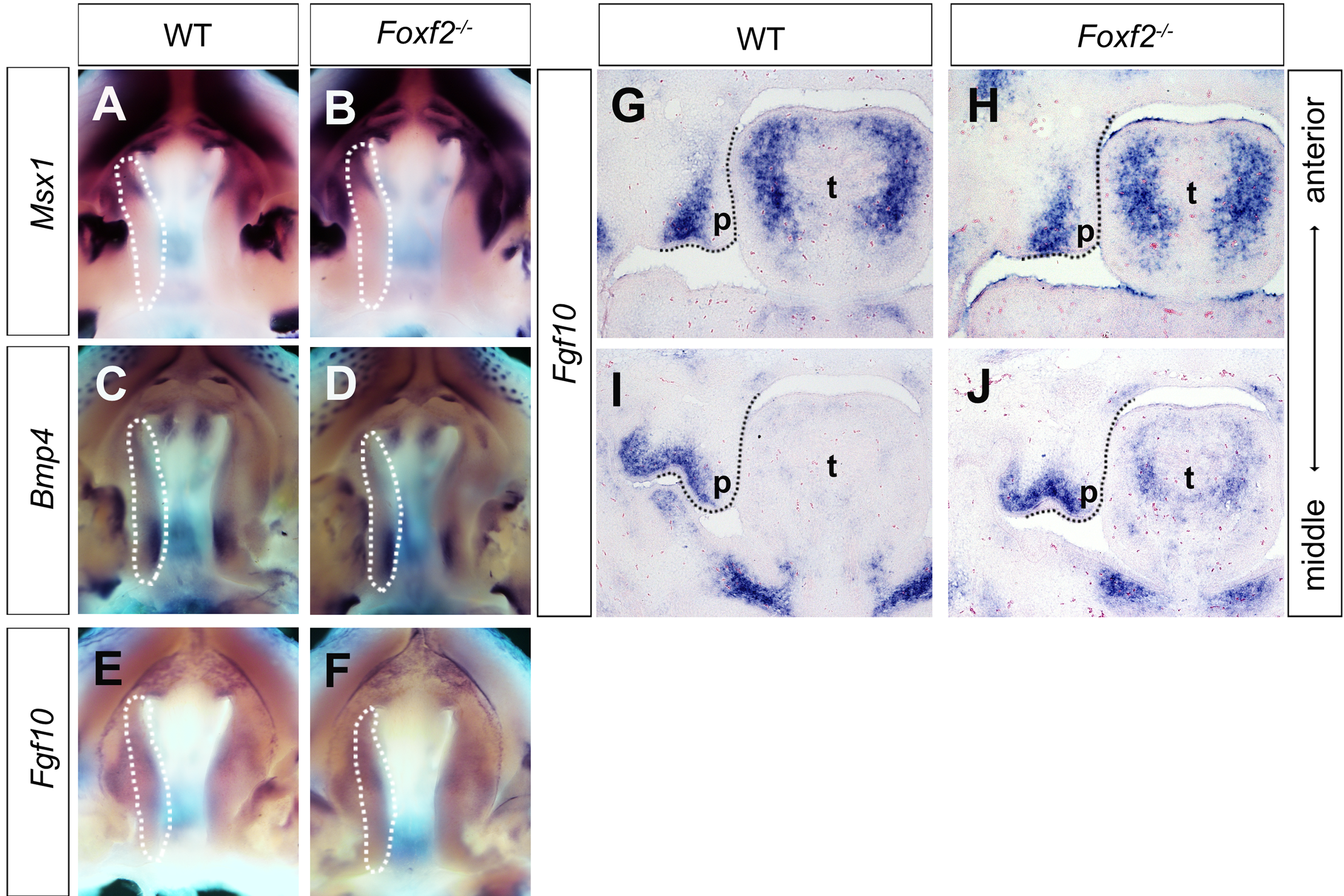

Supplement: S1 Fig — (A-F) Whole-mount in situ hybridization detection of Msx1 (A, B), Bmp4 (C, D), and Fgf10 (E, F) mRNAs in the developing palatal shelves in wildtype (A, C, E) and Foxf2-/- mutant (B, D, F) embryos at E13.5. White dash lines indicate the palate region. (G-J) Frontal sections showing expression of Fgf10 mRNA in the anterior (G, H) and middle (I, J) regions of the developing palate in wildtype (G, I) and Foxf2-/- mutant (H, J) embryos at E13.5. p, palatal shelf; t, tongue. (TIF) [file pgen.1005769.s003.tif]

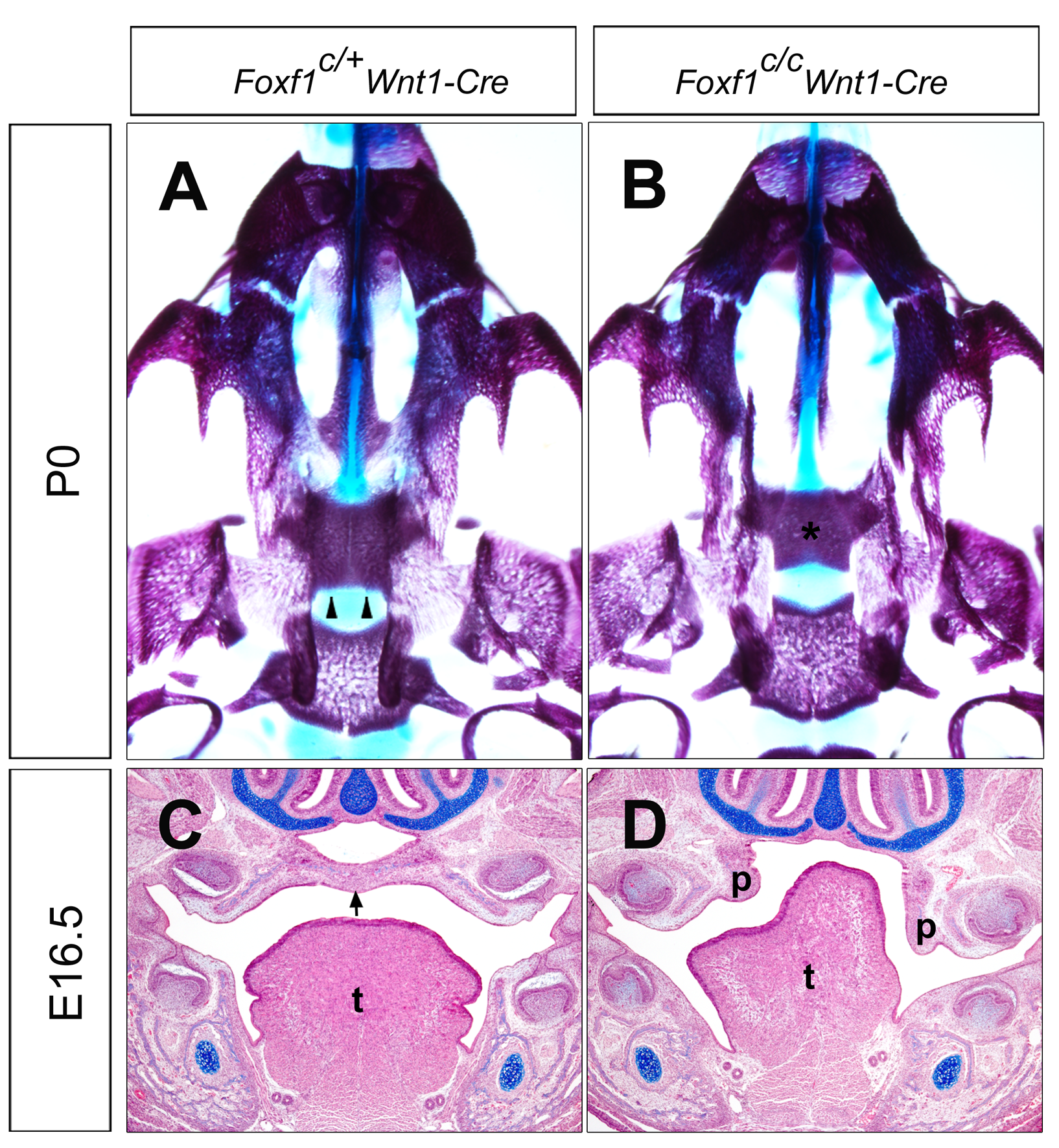

Supplement: S2 Fig — (A, B) Ventral view of stained skeletal preparations of Foxf1c/+Wnt1-Cre (A) and Foxf1c/cWnt1-Cre (B) neonatal skulls. Arrowheads indicate palatal processes of the palatine bones that have fused to each other in the Foxf1c/+Wnt1-Cre mice (A) but are absent in the Foxf1c/cWnt1-Cre mice, exposing the presphenoid bone (marked with an asterisk) underneath (B). (C, D) Representative frontal sections from developing palatal shelves of Foxf1c/+Wnt1-Cre (C), and Foxf1c/cWnt1-Cre (D) embryos, at E16.5. p, palatal shelf; t, tongue. (TIF) [file pgen.1005769.s004.tif]

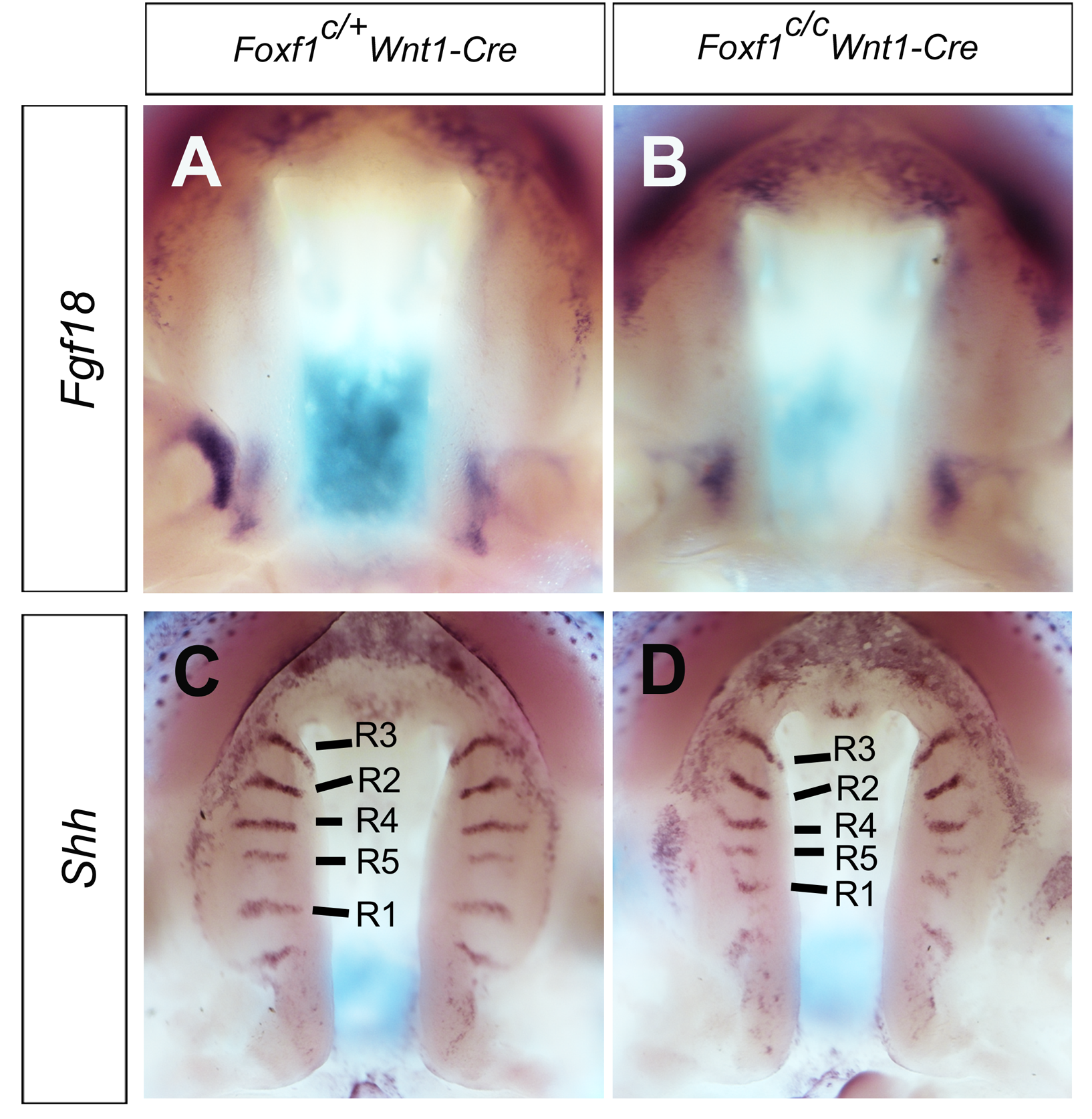

Supplement: S3 Fig — (A, B) Whole-mount in situ hybridization detection of Fgf18 mRNAs in the developing palatal shelves in Foxf1c/c (A) and Foxf1c/cWnt1-Cre mutant (B) embryos at E13.5. (C, D) Whole-mount in situ hybridization detection of Shh mRNAs in the developing palatal shelves in Foxf1c/c (C) and Foxf1c/cWnt1-Cre mutant (D) embryos at E13.5. (TIF) [file pgen.1005769.s005.tif]

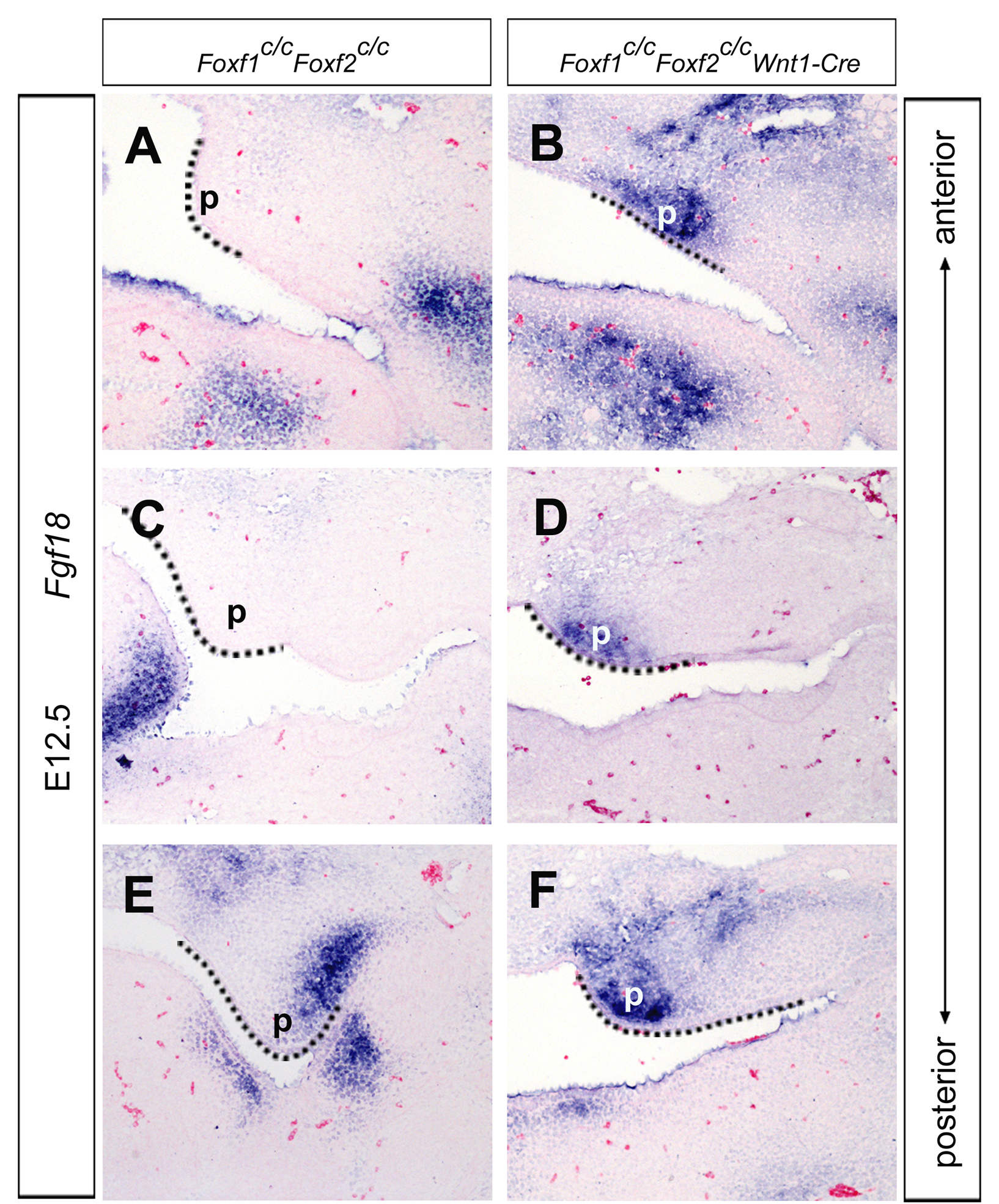

Supplement: S4 Fig — Frontal sections showing expression of Fgf18 mRNA in the anterior (A, B), middle (C, D) and posterior (E, F) regions of the developing palate in Foxf1c/cFoxf2c/c (A, C, E) and Foxf1c/cFoxf2c/cWnt1-Cre mutant (B, D, F) embryos at E12.5. p, palatal shelf. (TIF) [file pgen.1005769.s006.tif]

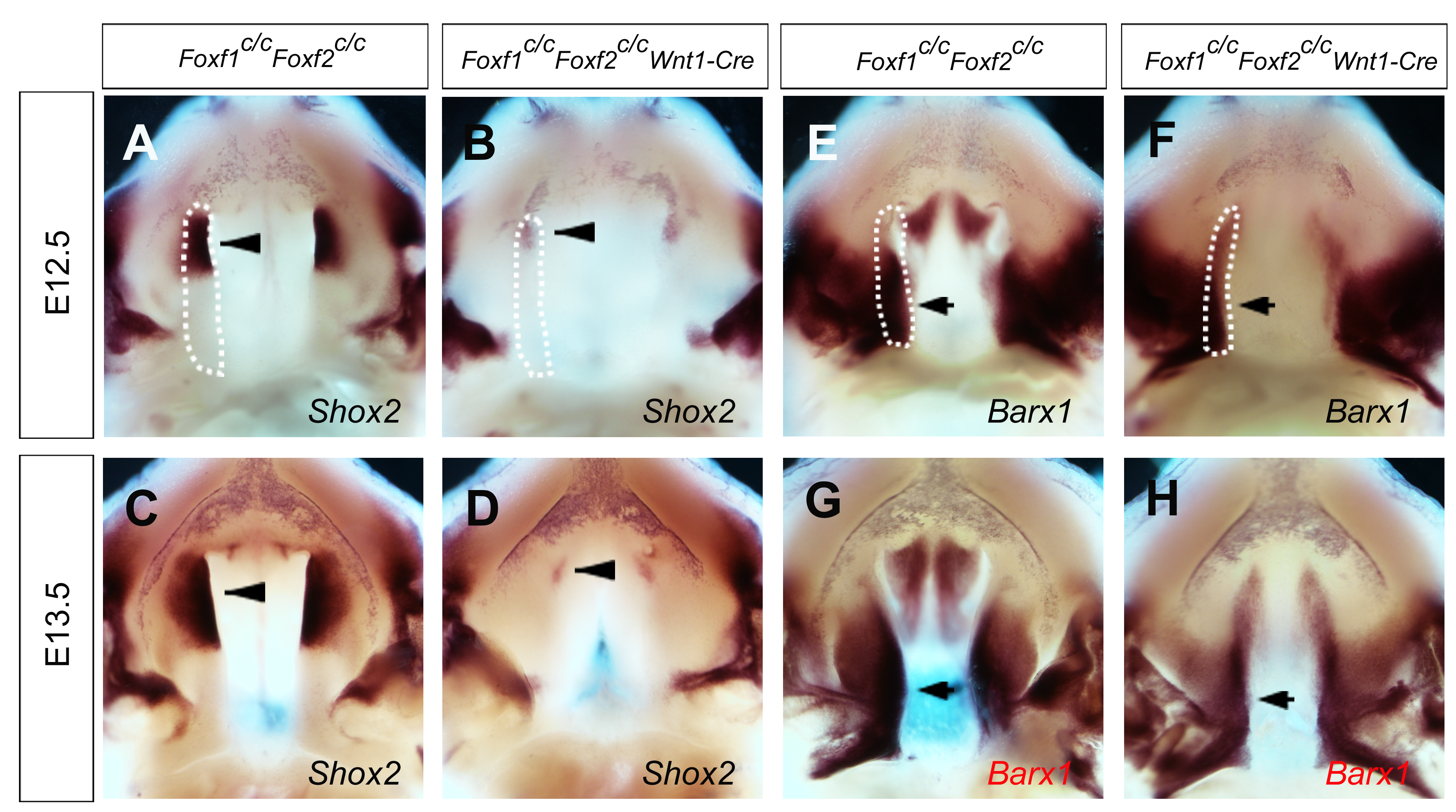

Supplement: S5 Fig — (A-D) Whole-mount in situ hybridization detection of Shox2 mRNAs in the developing palatal shelves in Foxf1c/cFoxf2c/c (A, C) and Foxf1c/cFoxf2c/cWnt1-Cre mutant (B, D) embryos at E12.5 (A, B) and E13.5 (C, D). (E-H) Whole-mount in situ hybridization detection of Barx1 mRNAs in the developing palatal shelves in Foxf1c/cFoxf2c/c (E, G) and Foxf1c/cFoxf2c/cWnt1-Cre mutant (F, H) embryos at E12.5 (E, F) and E13.5 (G, H). (TIF) [file pgen.1005769.s007.tif]
